# Nomogram development and external validation for predicting overall survival and cancer-specific survival in patients with primary retroperitoneal sarcoma: a retrospective cohort study

**DOI:** 10.1007/s12672-023-00804-1

**Published:** 2023-11-01

**Authors:** Jialiang Zheng, Aobo Zhuang, Xiaogang Xia, Fenglin Miao, Zhao Wang, Xu Kong, Yantao Ren, Yuan Ma, Zhenhang Lin, Weiqi Lu, Wengang Li

**Affiliations:** 1 Cancer Research Center, Department of Hepatobiliary Surgery, Xiang’an Hospital of Xiamen University, School of Medicine, Xiamen University, Xiamen, 361102 Fujian China; 2 Xiamen University Research Center of Retroperitoneal Tumor Committee of Oncology Society of Chinese Medical Association, Xiamen University, Xiamen, 361102 Fujian China; 3 Department of General Surgery, Zhongshan Hospital, Fudan University, Shanghai, 200000 China

**Keywords:** Retroperitoneal sarcoma, Survival, Prognostic factors, Prediction model, Risk screening, SEER

## Abstract

**Background:**

Primary retroperitoneal sarcoma (RPS) comprises over 70 histologic subtypes, yet there are limited studies that have developed prognostic nomograms for RPS patients to predict overall survival (OS) and cancer-specific survival (CSS). The objective of this study was to construct prognostic nomograms for predicting OS and CSS in RPS patients.

**Methods:**

We identified a total of 1166 RPS patients from the Surveillance, Epidemiology and End Results (SEER) database, and an additional 261 cases were collected from a tertiary cancer center. The study incorporated various clinicopathological and epidemiologic features as variables, and prediction windows for overall survival (OS) and cancer-specific survival (CSS) were set at 3, 5, and 7 years. Multivariable Cox models were utilized to develop the nomograms, and variable selection was performed using a backward procedure based on the Akaike Information Criterion. To evaluate the performance of the nomograms in terms of calibration and discrimination, we used calibration plots, coherence index, and area under the curve.

**Findings:**

The study included 818 patients in the development cohort, 348 patients in the internal validation cohort, and 261 patients in the external validation cohort. The backward procedure selected the following variables: age, French Federation of Cancer Centers Sarcoma Group (FNCLCC) grade, pre-/postoperative chemotherapy, tumor size, primary site surgery, and tumor multifocality. The validation results demonstrated that the nomograms had good calibration and discrimination, with C-indices of 0.76 for OS and 0.81 for CSS. Calibration plots also showed good consistency between the predicted and actual survival rates. Furthermore, the areas under the time-dependent receiver operating characteristic curves for the 3-, 5-, and 7-year OS (0.84, 0.82, and 0.78, respectively) and CSS (0.88, 0.88, and 0.85, respectively) confirmed the accuracy of the nomograms.

**Interpretation:**

Our study developed accurate nomograms to predict OS and CSS in patients with RPS. These nomograms have important clinical implications and can assist healthcare providers in making informed decisions regarding patient care and treatment options. They may also aid in patient counseling and stratification in clinical trials.

**Supplementary Information:**

The online version contains supplementary material available at 10.1007/s12672-023-00804-1.

## Introduction

Primary retroperitoneal sarcoma (RPS) is a rare malignancy, accounting for about 0.1% of adult cancers globally, and clinical estimates project an annual occurrence of 0.5 to 1 new case per 100,000 individuals [1]. RPS originates within the expansive retroperitoneal space, characterized by an absence of discernible symptoms during its initial stages. Clinical manifestations only become evident in the later stages when it encroaches upon or exerts pressure upon neighboring organs. At this juncture, the neoplasm often attains significant proportions and infiltrates adjacent structures, thus resulting in an unfavorable prognosis for the patient [2]. RPS comprises more than 70 different histologic subtypes [3], these types of pathology are further complicated by their variable presentation, behavior, and long-term outcomes, which emphasize the importance of accurate prognostic models to guide patient management. Surgical resection is the preferred treatment option for RPS, and it can significantly improve patient prognosis [4–7]. However, not all patients are suitable candidates for surgery due to factors such as metastasis or extensive tumor burden.

Due to the rarity of primary RPS and the diverse range of histological subtypes, predicting patient prognosis can be challenging. A nomogram is a valuable visual tool in clinical medicine. It provides personalized survival estimates based on individual patient characteristics, aiding doctors in understanding survival probabilities, assessing risk, and supporting treatment decisions. In clinical investigation, nomograms help identify suitable participants and enhance trial efficiency and reliability. They also facilitate patient and family understanding of survival probabilities and risks, improving trust and treatment plan adherence. In summary, nomograms significantly improve patient care and outcomes. Although several nomograms have been developed to predict the prognosis of RPS patients with surgical resection [8–13], none have been able to predict overall survival (OS) and cancer cause-specific survival (CSS) for all RPS patients. Therefore, it is crucial to identify effective prognostic models that can assess the OS and CSS of RPS patients with different pathological types.

We collected clinicopathological and epidemiological information of patients with primary retroperitoneal sarcoma (RPS) from the Surveillance, Epidemiology and End Results (SEER) database [14]. Additionally, we collected information on RPS patients from a tertiary cancer center. The SEER cohort was divided into a training cohort and an internal validation cohort, with additional data collected for the external validation cohort, and significant variables were selected through Cox proportional hazards regression. The ultimate aim was to develop nomograms that could accurately predict OS and CSS and perform effective risk stratification in RPS patients.

This study developed and validated nomograms for RPS patients to predict OS and CSS. These nomograms provide an accurate tool for clinicians in patient care and research, enabling them to assess disease progression risk and guide treatment decisions.

## Materials and methods

The study received ethical approval from the institutional review board of the South Hospital of the Zhongshan Hospital/Shanghai Public Health Clinical Center, Fudan University, Shanghai, China. Patient information used in the study was anonymized to protect their privacy. No ethical approval was required for the use of information in the SEER Program.

### Patients and definitions

**Fig. 1:**
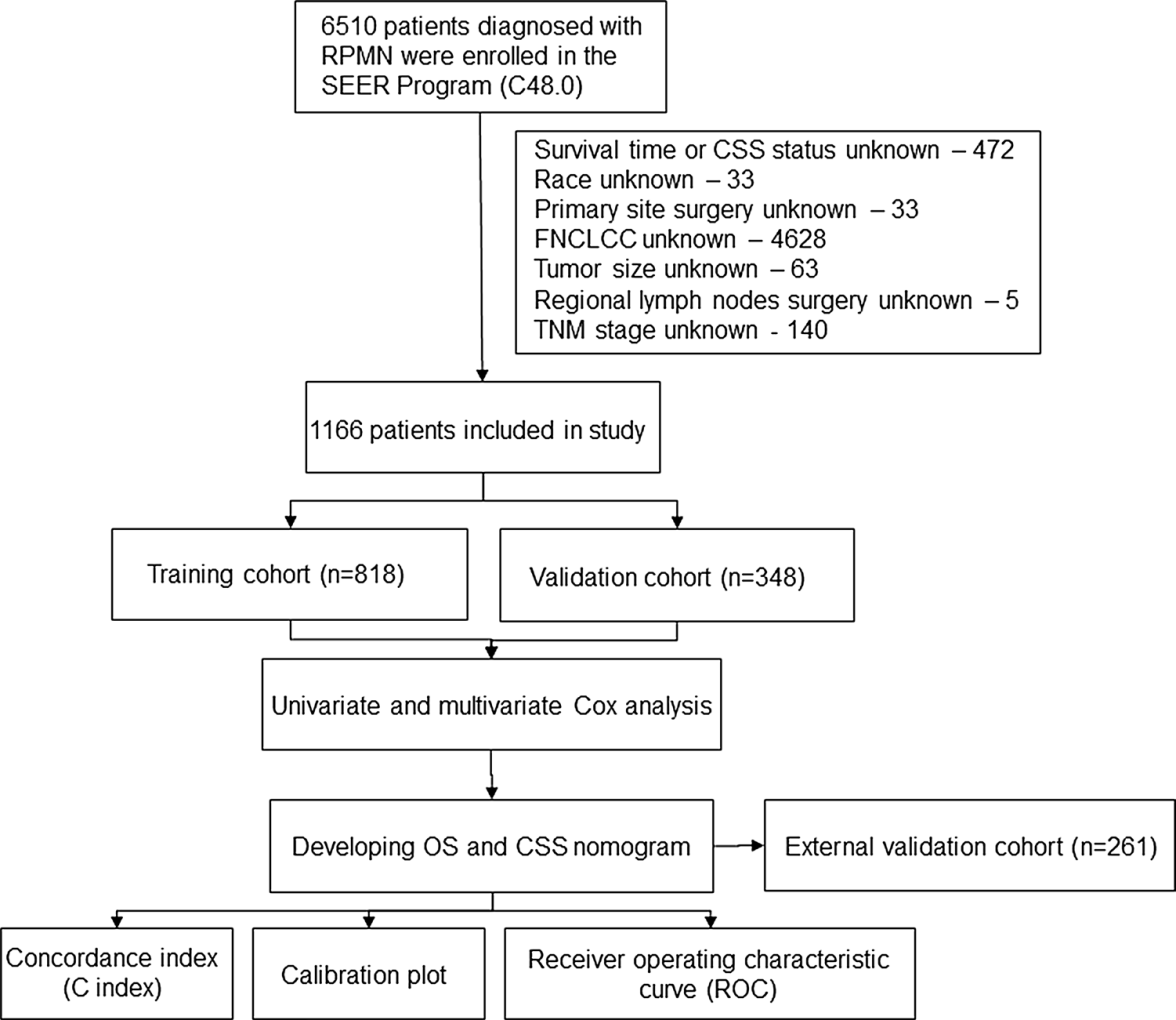
Patient data selection process. SEER: Surveillance, Epidemiology, and End Results

The SEER database is a widely recognized and respected source of information on cancer epidemiology and clinical characteristics. It provides comprehensive data on cancer diagnoses and survival for around 30% of the US population [14]. We identified patients with RPS from January 1, 2000, to December 31, 2019, in the SEER database using the International Classification of Diseases for Oncology, Third Edition (ICD-O-3) primary site code (C48.0) and behavior code (malignant), and cases with missing or unknown clinicopathological and epidemiological data were discarded (Fig. 1). We compiled clinicopathological and epidemiological data of the patients, including age, sex, race, ICD-O-3 pathology type, the French Federation of Cancer Centers Sarcoma Group (FNCLCC) grade, primary site surgery (no surgery of primary site and total surgical removal of primary site), regional lymph node surgery, tumor multifocality, tumor size, chemotherapy data, median household income, local population size and degree of urbanization, survival status and survival time. Tumor size was a combination of the fields “CS Tumor Size (2004–2015)” and “Tumor Size Summary (2016+)”, FNCLCC grade of patients was a combination of the fields “CS site-specific factor 1 (2004–2017 varying by schema)”, “Grade Pathological (2018+)” and “Grade Clinical (2018+)”, TNM stage was a combination of the fields “Derived AJCC T, N, M, 7th ed (2010–2015)”, “Derived SEER Combined T, N, M (2016–2017)” and “Derived EOD 2018T, N, M (2018+)”. Pathological types were classified as liposarcoma [ICD-O-3/WHO 2008 morphology codes: 8850, 8851, 8852, 8853, 8854, 8855, 8857, 8858, n = 689], leiomyosarcoma [8890, 8891, 8894, 8895, 8896, n = 356], fibrosarcoma [8810, 8811, 8813, 8814, 8815, n = 19] and others [n = 102]. The patient data collected from the tertiary cancer center also adhered to the same principles as mentioned above.

### Study design

We aimed to construct nomograms that predict patients OS and CSS prognosis at 3-, 5- and 7-year. Patient data from SEER was obtained using SEER*Stat software, version 8.4.0 (Surveillance Research Program, National Cancer Institute). Finally, a total of 1427 patients were included, 1166 patients were from SEER and 261 patients were from the tertiary cancer center. Patient data from SEER was randomly divided into a developing set [n = 818, 70.2%] and an internal validation set [n = 348, 29.8%], the patient data from the tertiary cancer center were used for external validation [n = 261]. X-tile software was used to calculate the optimal cutoff values for the conversion of continuous variables into categorical variables [15]. Age, tumor size, median household income, local population size, and degree of urbanization were converted to categorical variables. For OS, X-tile software divided age into three groups: ≤ 55 years, 56–78 years, and ≥ 79 years, tumor size was divided into two groups: ≤ 7.7 cm and > 7.7 cm; for CSS, age was divided into ≤ 54 years, 55–78 years and ≥ 79 years three groups, tumor size was divided into ≤ 10.4 cm and > 10.4 cm two groups. In both OS and CSS, the median household income of patients was divided into two groups: 0–54999 dollars and ≥ 55,000 dollars; population size and degree of urbanization in the patient’s place was divided into two groups [I: nonmetropolitan counties not adjacent to a metropolitan area, nonmetropolitan counties adjacent to a metropolitan area and counties in metropolitan areas of less than 250 thousand population; II: counties in metropolitan areas of 250,000 to 1 million population and counties in metropolitan areas greater than or equal to 1 million population]. Univariate and multivariate Cox regression analyses were performed based on the developing set, variables with P < 0.05 in univariate Cox regression analysis were included in multivariate Cox regression analysis. To develop the two nomograms, we used multivariable Cox regression analysis using a backward procedure with Akaike information criterion (AIC) as the variables selection criterion for OS and CSS models [16]. Internal and external validation of the OS nomogram and internal validation of the CSS nomogram were performed, and concordance indices (C index) and calibration plots were used to measure discrimination and calibration ability. Furthermore, the accuracy of the nomograms for 3-, 5- and 7-year survival prediction were compared with the TNM staging system to demonstrate by using the area under (AUC) the receiver operating characteristic curve (ROC).

### Statistical analysis

Statistical analysis was performed using R software, version 4.0.3 (R Foundation for Statistical Computing). Variable selection based on AIC was implemented using the MASS R package [17], potentially prognostic factors, age, sex, race and ethnicity, tumor size, histopathological type, FNCLCC grade, tumor multifocality, surgery of primary site, surgery of regional lymph nodes, pre-/postoperative chemotherapy, median household income, local population size and degree of urbanization, were all included. Multivariable Cox regression analysis was performed to calculate the hazard ratios, two-sided Wald test P values, and 95% confidence intervals of the selected variables and build the nomograms. A risk score of a single patient was calculated using the survival R package and the median was used as a cutoff value for high-risk and low-risk groups [18]. Statistical comparison of survival curves was using a log-rank test. The internal and external validation sets were applied in the model built using the developing set. The bootstrap method was applied for 1000 iterations of computation.

## Results

### Patient characteristics

The baseline clinicopathological and epidemiological characteristics of the 1427 patients with RPS (mean [SD] age, 60.2 [15.0] years) were shown in Table 1, and the characteristics were well balanced between the developing set and the validation set.

**Table 1:** Demographic, clinical, and pathologic characteristics according to datasets

Characteristic	Developing set		Internal validation set		Total		External validation set
	No	%	No	%	No	%	No
Total	818	70.2	348	29.8	1166	100	
Age, years (OS/CSS)							
≤ 55/ ≤ 54	263/239	22.6/20.5	103/97	8.8/8.3	366/336	31.4/28.8	126
56–78/55–78	468/492	40.1/42.3	207/213	17.8/18.3	675/705	57.9/60.6	125
≥ 79	87	7.5	38	3.3	125	10.8	10
Tumor size, cm (OS/CSS)							
≤ 7.7/ ≤ 10.4	140/230	12.0/19.7	72/113	6.2/9.7	212/343	18.2/29.4	48
> 7.7/ > 10.4	678/588	58.1/50.4	276/235	23.7/20.2	954/823	81.8/70.6	213
Sex							
Female	439	37.7	180	15.4	619	53.1	
Male	379	32.5	168	14.4	547	46.9	
Histology							
Liposarcoma	487	41.8	202	17.3	689	59.1	159
Leiomyosarcoma	254	21.8	102	8.7	356	30.5	41
Fibrosarcoma	13	1.1	6	0.5	19	1.6	7
Other	64	5.5	38	3.3	102	8.8	54
Race							
White	630	54.0	276	23.7	906	77.7	0
Black	94	8.1	33	2.8	127	10.9	0
Other	94	8.1	39	3.3	133	11.4	261
FNCLCC grade							
I	239	20.5	103	8.8	342	29.3	95
II	273	23.4	116	9.9	389	33.3	85
III	306	26.2	129	11.1	435	37.3	81
Multifocality							
No	580	49.7	244	20.9	824	70.6	238
Yes	238	20.4	104	8.9	342	29.3	23
Surgery primary site							
No	44	3.8	27	2.3	71	6.1	0
Yes	774	66.4	321	27.5	1095	93.9	261
Surgery regional lymph							
No	552	47.3	231	19.8	783	67.1	
Yes	266	22.8	117	10.0	383	32.8	
Pre-/postoperative chemotherapy							
No/unknown	665	57.0	280	24.0	945	81.0	234
Yes	153	13.1	68	5.8	221	18.9	27
Median household income, dollars							
0–54999	156	13.4	75	6.4	231	19.8	
≥ 55,000	662	56.8	273	23.4	935	80.2	
Rural–Urban							
I	142	12.2	58	5.0	200	17.2	
II	676	58.0	290	24.9	966	82.9	
T status							
T1	64						
T2	633						
T3	36						
T4	85						
N status							
N0	787						
N1	31						
M status							
M0	754						
M1	64						

*FNCLCC* the French Federation of Cancer Centers Sarcoma Group, *Rural–Urban* I: nonmetropolitan counties not adjacent to a metropolitan area, nonmetropolitan counties adjacent to a metropolitan area, and counties in metropolitan areas of less than 250 thousand population; II: counties in metropolitan areas of 250,000 to 1 million population and counties in metropolitan areas greater than or equal to 1 million population

Among the study population, 619 (43.4%) patients were female, and 547 (38.3%) were male. The median age was 60 (range, 0–95) years. The median tumor size was 139.5 (range, minimum: 11, maximum: > 989) mm. The median follow-up time was 29 (range, 1–140) months. The median OS and CSS were 56.21 (IQR: 47.20–71.10) and 67.54 (IQR: 61.01–78.15) months, respectively. Patients diagnosed with liposarcoma, leiomyosarcoma, fibrosarcoma and other pathological types were 848 (59.4%), 397 (27.8%), 26 (1.9%) and 156 (10.9%) respectively. Race and ethnicity of patients were divided into 3 categories: White (906, 63.5%), Black (127, 8.9%), and others (394, 27.6%).

### Variable selection and multivariable Cox analysis

Results of univariate and multivariate Cox regression analysis were displayed in Table 2, P < 0.05 was considered significant. Based on AIC, age, FNCLCC grade, chemotherapy, tumor size, primary site surgery, and tumor multifocality were identified as independent risk factors for OS and CSS. The results of the final OS and CSS multivariate Cox regression models were displayed in Table 3. The multivariable Cox analysis of the developing set found that older age (56–78 years vs ≤ 55 years, hazard ratio [HR], 1.49; 95% confidence interval [CI] 1.14–1.96; P < 0.01; ≥ 79 years vs ≤ 55 years, HR, 3.00; 95% CI 2.06–4.38; P < 0.001), higher FNCLCC grade (grade II vs grade I, HR, 2.11; 95% CI 1.48–3.02; grade III vs grade I, HR, 3.95; 95% CI 2.85–5.49; both P < 0.001), pre-/postoperative of chemotherapy (yes vs no, HR, 2.01; 95% CI, 1.53–2.64; P < 0.001), tumor size > 7.7 cm (> 7.7 cm vs ≤ 7.7 cm, HR, 3.06; 95% CI 1.96–4.79; P < 0.001), no surgery operated on primary site (no vs yes, HR, 3.35; 95% CI 2.23–5.04; P < 0.001) were independent risk factors for worse OS, and older age (55–78 years vs ≤ 54 years, HR, 1.43; 95% CI 1.03–1.98; P < 0.05; ≥ 79 years vs ≤ 54 years, HR, 2.54; 95% CI 1.54–4.19; P < 0.001), higher FNCLCC grade (grade II vs grade I, HR, 2.69; 95% CI 1.67–4.34; grade III vs grade I, HR, 5.30; 95% CI 3.39–8.28; both P < 0.001), pre-/postoperative of chemotherapy (yes vs no, HR, 1.93; 95% CI 1.39–2.69; P < 0.001), tumor size > 10.4 cm (> 10.4 cm vs ≤ 10.4 cm, HR, 2.60; 95% CI 1.74–3.88; P < 0.001), no surgery operated on primary site (no vs yes, HR, 4.58; 95% CI 2.90–7.23; P < 0.001), single neoplasm (single vs multiple, HR, 4.419; 95% CI 2.67–7.31; P < 0.001) were independent risk factors for worse CSS. Median household income and local population size and degree of urbanization were excluded based on AIC in both multivariable Cox models.

**Table 2:** Univariable and multivariable Cox regression for analyzing the risk factors affecting overall and cancer-specific survival

Characteristics	OS						CSS					
	Univariable			Multivariable			Univariable			Multivariable		
	HR	95%CI	P	HR	95%CI	P	HR	95%CI	P	HR	95%CI	P
Age, years (OS/CSS)												
≤ 55/ ≤ 54	–	–	–	–	–	–	–	–	–	–	–	–
56–78/55–78	1.38	1.05–1.81	< 0.05	1.51	1.15–1.99	< 0.01	1.20	0.87–1.65	0.28	1.48	1.06–2.06	< 0.05
≥ 79	2.80	1.96–4.02	< 0.001	3.06	2.09–4.47	< 0.001	1.85	1.15–2.98	< 0.05	2.68	1.61–4.46	< 0.001
Tumor size, cm (OS/CSS)												
≤ 7.7/ ≤ 10.4	–	–	–	–	–	–	–	–	–	–	–	–
> 7.7/ > 10.4	3.00	1.92–4.68	< 0.001	3.00	1.89–4.76	< 0.001	2.35	1.58–3.48	< 0.001	2.87	1.85–4.45	< 0.001
Sex												
Female	–	–	–	–	–	–	–	–	–	–	–	–
Male	1.62	0.92–1.46	0.20				1.02	0.77–1.35	0.91			
Histology												
Liposarcoma	–	–	–	–	–	–	–	–	–	–	–	–
Leiomyosarcoma	1.09	0.84–1.41	0.52	0.92	0.69–1.22	0.56	1.20	0.88–1.64	0.25	1.14	0.80–1.62	0.48
Fibrosarcoma	1.56	0.73–3.32	0.25	0.87	0.40–1.89	0.73	1.37	0.50–3.71	0.54	0.56	0.20–1.56	0.27
Other	2.07	1.44–2.99	< 0.001	1.14	0.78–1.67	0.50	2.00	1.26–3.17	< 0.01	1.23	0.76–2.00	0.39
Race												
White	–	–	–	–	–	–	–	–	–	–	–	–
Black	1.16	0.82–1.65	0.40				1.23	0.81–1.89	0.33			
Other	1.05	0.74–1.50	0.77				1.44	0.98–2.11	0.07			
FNCLCC												
I	–	–	–	–	–	–	–	–	–	–	–	–
II	2.11	1.49–2.98	< 0.001	2.15	1.49–3.13	< 0.001	2.80	1.76–4.45	< 0.001	2.65	1.61–4.36	< 0.001
III	4.18	3.04–5.74	< 0.001	3.90	2.78–5.48	< 0.001	5.65	3.68–8.70	< 0.001	5.17	3.27–8.17	< 0.001
Multifocality												
No	–	–	–	–	–	–	–	–	–	–	–	–
Yes	0.83	0.65–1.07	0.16				0.20	0.12–0.33	< 0.001	0.22	0.13–0.37	< 0.001
Surgery primary												
No	–	–	–	–	–	–	–	–	–	–	–	–
Yes	0.22	0.15–0.32	< 0.001	0.30	0.20–0.45	< 0.001	0.17	0.11–0.26	< 0.001	0.21	0.14–0.34	< 0.001
Surgery lymph												
No	–	–	–	–	–	–	–	–	–	–	–	–
Yes	0.91	0.71–1.17	0.46				0.90	0.66–1.22	0.49			
Pre-/postoperative												
No/unknown	–	–	–	–	–	–	–	–	–	–	–	–
Yes	2.73	2.12–3.51	< 0.001	2.00	1.52–2.63	< 0.001	3.17	2.35–4.27	< 0.001	1.82	1.30–2.56	< 0.001
Median household income, dollars												
0–54999	–	–	–	–	–	–	–	–	–	–	–	–
≥ 55,000	0.72	0.55–0.95	< 0.05	0.83	0.63–1.09	0.18	0.72	0.51–1.00	0.048	0.92	0.66–1.29	0.62
Rural–Urban												
I	–	–	–	–	–	–	–	–	–	–	–	–
II	0.86	0.64–1.15	0.31				0.83	0.58–1.18	0.30

**Table 3:** Results of the final multivariable cox models (after the AIC-based backward selection) for OS and CSS

Variable	OS			CSS		
	HR	95% CI	P	HR	95% CI	P
Age, years (OS/CSS)						
≤ 55/ ≤ 54	–	–	–	–	–	–
56–78/55–78	1.49	1.14 to 1.96	< 0.01	1.43	1.03 to 1.98	0.033
≥ 79	3.00	2.06 to 4.38	< 0.001	2.54	1.54 to 4.19	< 0.001
Tumor size, cm (OS/CSS)						
≤ 7.7/ ≤ 10.4	–	–	–	–	–	–
> 7.7/ > 10.4	3.06	1.96 to 4.79	< 0.001	2.60	1.74 to 3.88	< 0.001
FNCLCC grade						
I	–	–	–	–	–	–
II	2.11	1.48 to 3.02	< 0.001	2.69	1.67 to 4.34	< 0.001
III	3.95	2.85 to 5.49	< 0.001	5.30	3.39 to 8.28	< 0.001
Multifocality						
No	–	–	–	–	–	–
Yes	–	–	–	0.23	0.14 to 0.37	< 0.001
Surgery primary site						
No	–	–	–	–	–	–
Yes	0.30	0.20 to 0.45	< 0.001	0.22	0.14 to 0.35	< 0.001
Pre-/postoperative chemotherapy						
No/unknown	–	–	–	–	–	
Yes	2.01	1.53 to 2.64	< 0.001	1.93	1.39 to 2.69	− < 0.001

*FNCLCC* the French Federation of Cancer Centers Sarcoma Group, *Rural–Urban* I: nonmetropolitan counties not adjacent to a metropolitan area, nonmetropolitan counties adjacent to a metropolitan area, and counties in metropolitan areas of less than 250 thousand population; II: counties in metropolitan areas of 250,000 to 1 million population and counties in metropolitan areas greater than or equal to 1 million population

### Nomogram development and validation

**Fig. 2:**
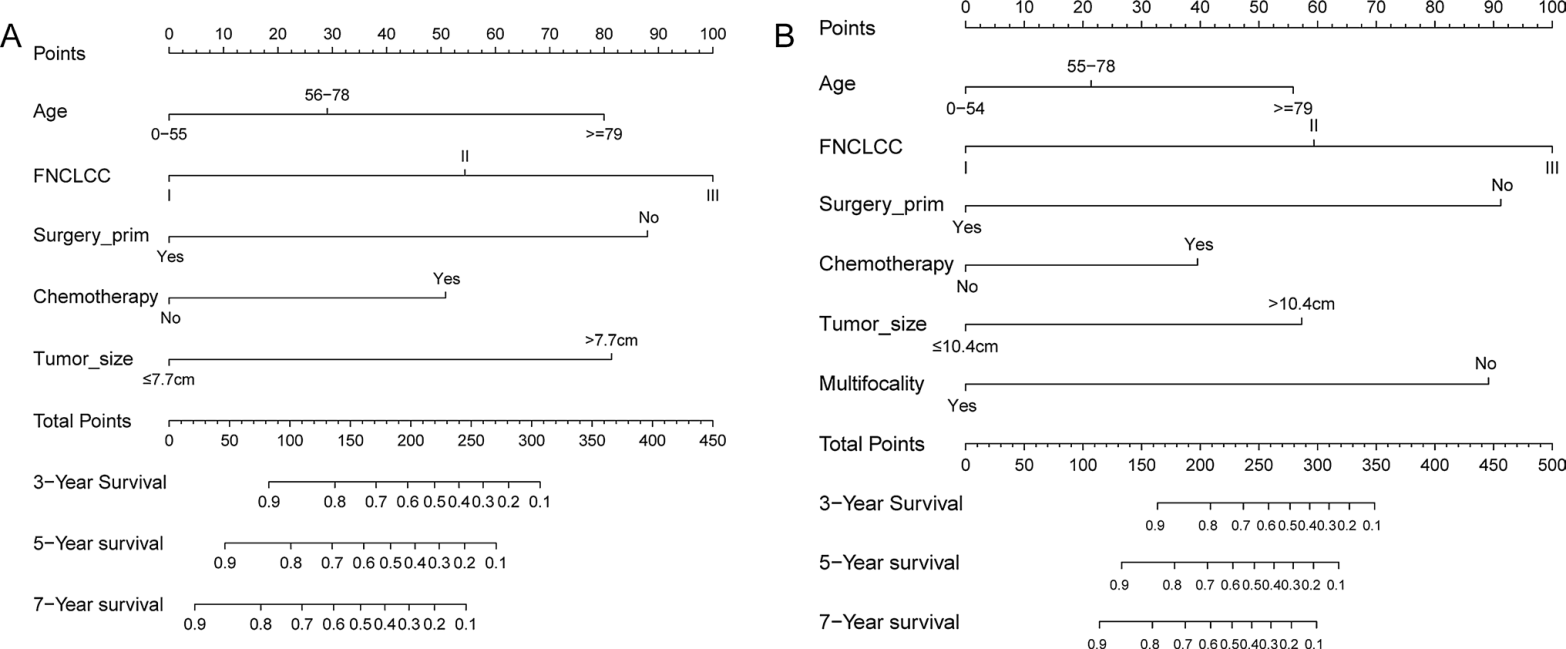
Nomograms for Predicting OS (**A**) and CSS (**B**) Based on Results of Multivariable Cox Analysis of Developing Set

Based on the developing sets, nomogram models for OS and CSS were constructed respectively by including associated factors, according to the multivariate Cox regression model (Fig. 2). FNCLCC grade had the greatest significance and can contribute a maximum of 100 points in both OS and CSS nomograms.

The OS nomogram was internally and externally validated and the CSS nomogram was only internally validated due to incomplete external validation data. The C indices for OS nomogram (developing set: 0.76 [95% CI 0.69–0.83]; internal validation set: 0.76 [95% CI 0.66–0.86]; external validation set: 0.69 [95% CI 0.58–0.80]; whole set: 0.75 [95% CI 0.70–0.80]) and CSS nomogram (developing set: 0.81 [95% CI 0.74–0.87]; internal validation set: 0.80 [95% CI 0.70–0.90]; whole set: 0.80 [95% CI 0.75–0.85]) were higher than that of TNM staging system (OS: developing set, 0.61 [95% CI 0.54–0.67]; internal validation set, 0.60 [95% CI 0.50–0.70]; whole set: 0.60 [95% CI 0.55–0.66]; CSS: developing set, 0.60 [95% CI 0.53–0.67]; internal validation set, 0.55 [95% CI 0.45–0.65]; whole set: 0.58 [95% CI 0.53–0.64]), which demonstrated that the models had good discrimination ability.

**Fig. 3:**
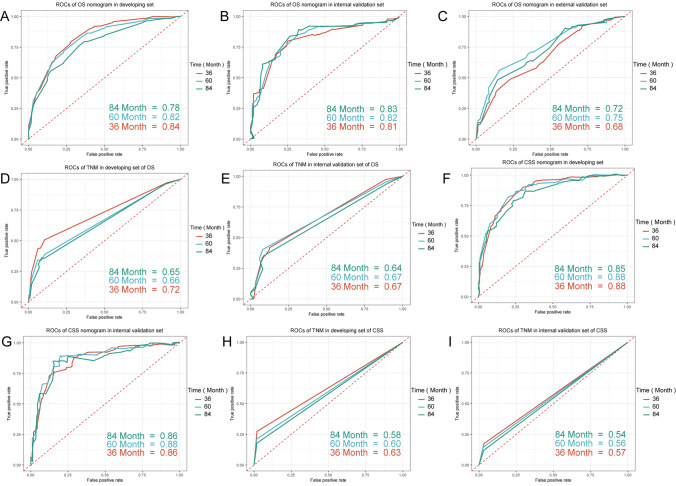
ROC Curves Evaluated the Predictive Ability of the Nomograms (**A**–**C**, **F**, **G**) and TNM Staging System (**D**, **E**, **H**, **I**). **A**–**C** nomogram for predicting OS ROCs in developing set, internal validation set, and external validation set respectively; **D**, **E** TNM staging system for predicting OS ROCs in developing set and internal validation set; **F**, **G** nomogram for predicting CSS ROCs in developing set and internal validation set; **H**, **I** TNM staging system for predicting CSS ROCs in developing set and internal validation set

Furthermore, we compared the predictive ability of the nomograms and TNM staging system through the AUC values of 3-, 5- and 7-year OS rates and CSS rates (Fig. 3). The AUC values of the nomogram for predicting 3-, 5- and 7-year OS were 0.84, 0.82, and 0.78 in the developing set, 0.81, 0.82, and 0.83 in the internal validation set, and 0.68, 0.75, and 0.72 in the external validation set, respectively, while for the TNM staging system, the AUCs were 0.72, 0.66 and 0.65 in the developing set, and 0.67, 0.67, and 0.64 in the internal validation set. The AUCs of the nomogram for predicting 3-, 5- and 7-year CSS were 0.88, 0.88, and 0.85 in the developing set, and 0.86, 0.88, and 0.86 in the internal validation set, respectively, while for the TNM staging system, the AUCs were 0.63, 0.60 and 0.58 in the developing set, and 0.57, 0.56, and 0.54 in the internal validation set.

**Fig. 4:**
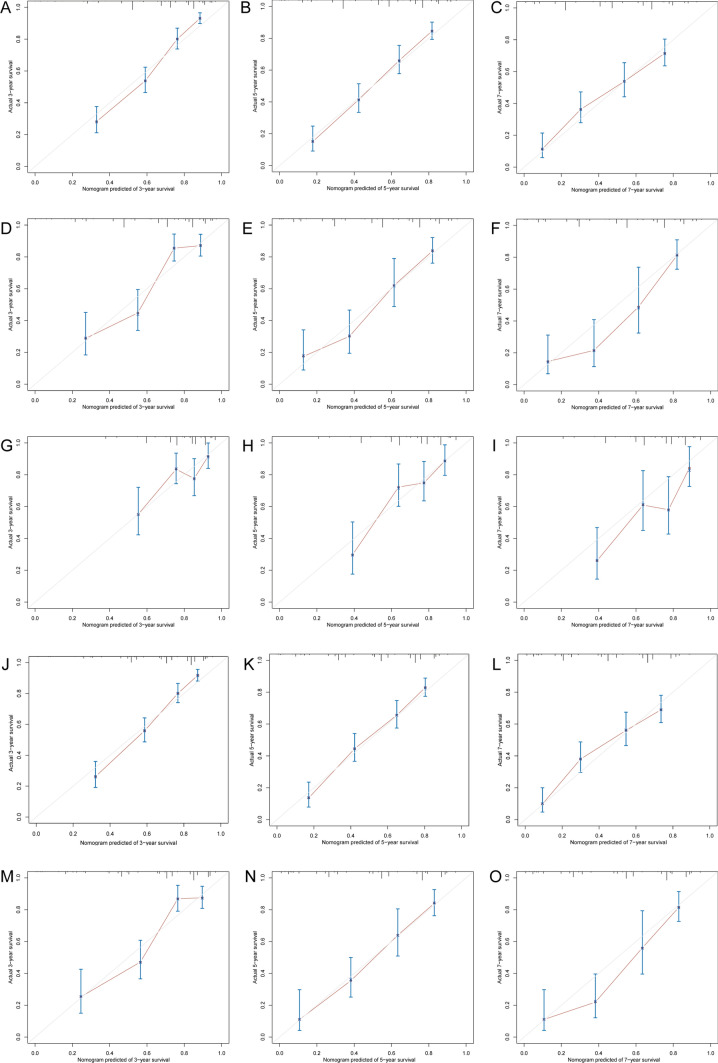
Calibration Plots for Estimating OS (**A**–**I**) and CSS (**J**–**O**) Survival Probability at 3, 5, and 7 Years. **A**–**C** Calibration plots in the OS developing set; **D**–**F** Calibration plots in the OS internal validation set; **G**–**I** Calibration plots in the OS external validation set; **J**–**L** Calibration plots in the CSS developing set; **M**–**O** Calibration plots in the CSS internal validation

**Fig. 5:**
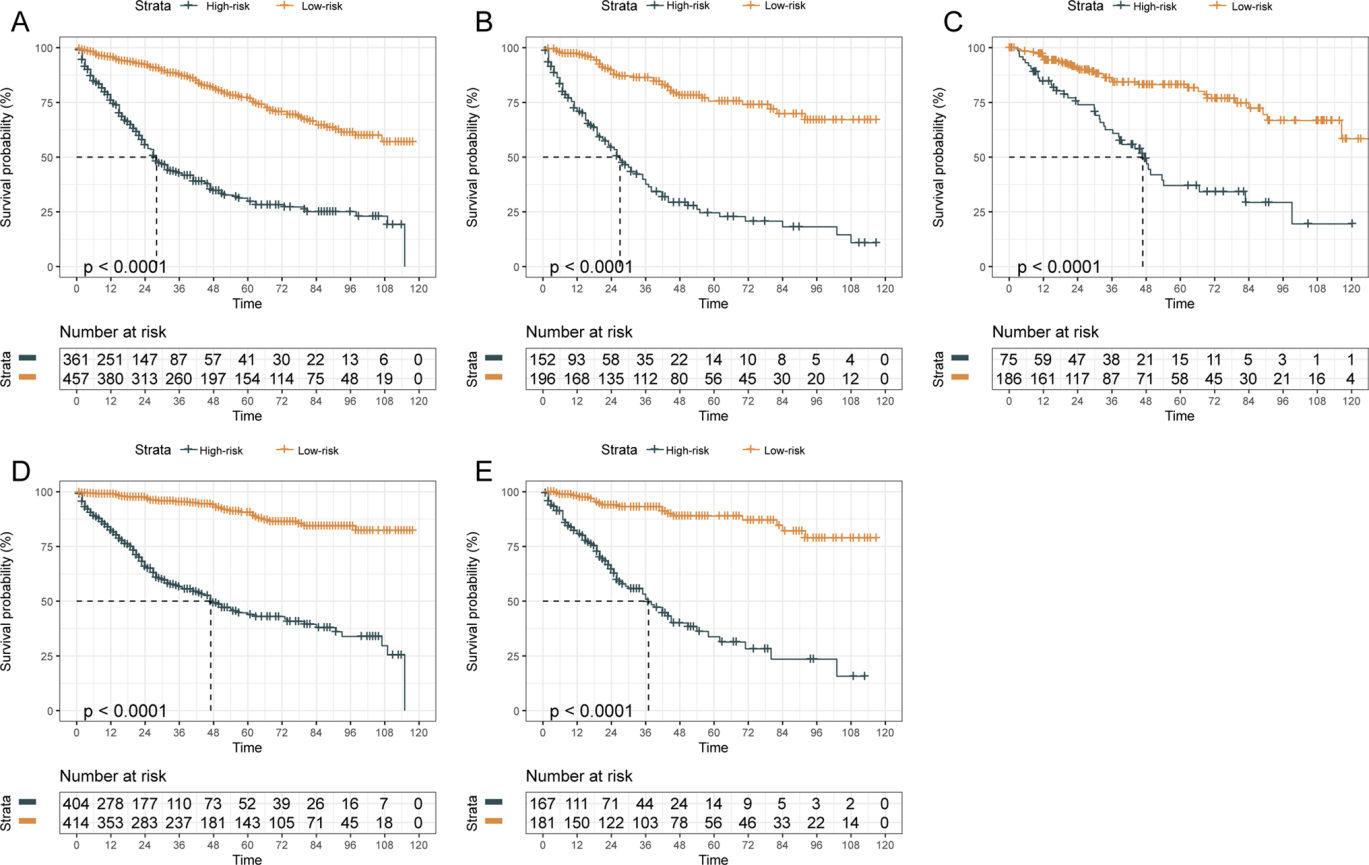
Kaplan–Meier OS (**A**–**C**) and CSS (**D**–**E**) Curves of Different Risk Groups for RPS Patients Based on Risk Group Stratification in Each Cohort. **A** Kaplan–Meier OS Curves of developing set; **B** Kaplan–Meier OS Curves of internal validation set; **C** Kaplan–Meier OS Curves of external validation set; **D** Kaplan–Meier CSS Curves of developing set; **E** Kaplan–Meier CSS Curves of the internal validation set

The calibration plots of the nomograms showed good consistency between the nomogram predicted and actual survival in the developing sets and validation sets (Fig. 4). Using the median risk score 0.91 and 0.33 as the cutoff values for OS and CSS models, respectively, the patients from each cohort were stratified into high-risk and low-risk groups. The OS and CSS curves from each dataset for the different risk groups were significantly different (log-rank P < 0.001 in each dataset, Fig. 5). The same conclusion was obtained for the validation sets.

## Discussion

Most patients with RPS have grave prognoses with a high recurrence rate, yet their life expectancy remains greatly various [3, 19], which makes survival difficult to assess. As a rare tumor, there are many pathological types of RPS, the most common pathological types, such as liposarcoma and leiomyosarcoma, have been studied more frequently [20–28], while other rare pathological types have been less studied. Based on the large global population, even rare pathological types deserve equal attention. In this population-based study, we constructed two nomograms for primary RPS patients, one for predicting OS and the other for predicting CSS. The prediction models were constructed based on the variables which were a combination of clinicopathological and epidemiological features, finally, age, tumor size, FNCLCC grade, primary site surgery, and chemotherapy were included in the OS nomogram, and tumor multifocality was additionally added in the CSS nomogram.

Recent studies have shown that higher income may mean lower mortality rates and better prognosis for patients [29, 30]. We were interested in the effect of household income and local population size, and degree of urbanization, which could partly represent the level of medical care in medical centers, on RPS patient outcomes. We additionally included them as variables in the constructed models, and the results showed that neither of them was an independent factor affecting patient outcomes in the models constructed in this study (Additional file 1: Table S1). We thought the reason was that the treatment options for RPS were still very limited. In addition, we found that based on the current histopathological classification system, pathological type was not the most representative prognostic factor affecting OS and CSS of RPS patients, in fact, in RPS, there are histological subtypes that exhibit relatively aggressive behavior and others that display relatively indolent characteristics. Their common feature is the invasion of surrounding organs. However, the treatment approaches for different histological subtypes of retroperitoneal sarcoma are essentially the same, with surgical resection being the preferred option. Resection of RPS often involves the pursuit of negative surgical margins [31] and may involve extensive multi-organ resection [1, 32]. Therefore, regardless of the histological subtype, the treatment for patients with RPS involves tumor removal along with partial organ resection. In fact, existing studies have shown that histological subtypes have a significant predictive effect on the recurrence of RPS, but the treatment strategies of different centers do not affect OS of well-differentiated liposarcoma [19]. Hence, the patient’s prognosis is likely more correlated with the treatment approach rather than the histopathological type. Thus, before the emergence of new histopathological subtype-specific treatment modalities, it may be unnecessary to add it as predictors of survival prognosis of RPS patients. However, there is no doubt that the histological subtype should be an important consideration in non-surgical treatments, such as radiotherapy, chemotherapy, targeted therapy, and immunotherapy, which have been extensively studied in other cancers such as breast cancer and lung cancer. The role of radiotherapy in the treatment of retroperitoneal sarcoma remains controversial, and recent studies have indicated the benefits of preoperative radiotherapy for certain specific histological subtypes of RPS [33, 34]. Adjuvant chemotherapy after surgery has been associated with worse OS in RPS, but there is a trend of improved OS with adjuvant chemotherapy for certain specific subtypes such as spindle cell, giant cell, and synovial sarcoma [35]. Currently, there is still insufficient research on the underlying mechanisms of RPS and other non-surgical treatment modalities in clinical studies. Therefore, overall, the treatment options for RPS remain relatively limited. Related clinical trials are currently underway and the results will be promising [4, 36, 37].

The most influential factor was FNCLCC grade. FNCLCC grade defines into three grades and contains tumor differentiation, mitotic count score, and tumor necrosis [38], which largely indicate the degree of tumor differentiation and reflect the pathological types of tumors to a certain extent. Therefore, we used FNCLCC grade to reflect pathological types and indicate their degree of malignancy, which also avoids the influence of collinearity between FNCLCC and histopathological types. The multifocality of the tumor in the CSS nomogram was contrary to the findings of previous studies [9, 19], which we believed may be due to the fact that multifocal tumors tend to be enlarged for resection, which in turn led to a better prognosis [1, 39–44]. Pre-/postoperative chemotherapy was identified as a risk factor, this was consistent with previous research [45, 46], we believe that chemotherapy should not be routinely administered for retroperitoneal sarcoma patients and that the chemotherapy regimen should be tailored to the specific histological type, pending validation in clinical trials.

Compared with other complex predictors, which were specific and highly correlated with the level of medical care in medical centers [47], the predictive factors included in the nomograms developed in this study were easily accessible, moreover, nomograms were developed using American cohort and still showed good accuracy in an Asian cohort. Thus, they have better fault tolerance and broad applicability and could minimize the impact of medical level differences among different medical centers on the predicted results. For RPS, surgery was the only possible way to cure patients [39, 48, 49], however, due to its extremely complex pathogenesis and tumor biological behavior, for example, the tumor load is large or the tumor involves important organs, some patients were not suitable for surgical treatment [50, 51]. To our knowledge, the current study was the first that constructed prognostic nomograms that could be applied to all patients with primary retroperitoneal malignancy with or without surgical treatment. In addition, the treatment strategies for high-risk groups of CSS were very limited in RPS. The nomogram could identify high-risk patients and help design specific clinical trials, which may have guiding significance for the treatment of RPS. At present, nomograms for RPS rely on variables derived from epidemiological and clinical-pathological indicators. To date, there have been no studies incorporating multi-omics features, such as transcriptomics, proteomics, metabolomics, or radiomics, into the construction of RPS nomograms. Given the ongoing technological advancements and the refinement of techniques such as single-cell sequencing and spatial transcriptomics, it becomes imperative to contemplate the integration of these multi-omics data for future prognostic analysis in RPS patients.

The predictive ability of nomograms has been verified both internally and externally, they have good discrimination ability, and C indices and calibration plots confirmed that. Compared with the TNM staging system, they have better performance. They would be beneficial to patient counseling and should be used in clinical practice. They could also be used to design clinical trials for patients with RPS, accurately stratify patients at risk and identify patients eligible for trials of novel therapies, and facilitate the development of specific clinical trials.

### Limitations

This study has several limitations that must be acknowledged. First, this is a retrospective cohort study, so there may be unavoidable selection bias, to address this limitation, we included relatively large cohort and divided it into a training set and a validation set by random grouping to construct the nomograms and validated internally and externally, these may reduce the bias caused by the retrospective data analysis. Second, we used external data from a single center for validation, hence, we hope to have our findings replicated in the future with large-scale data in other centers to validate the applicability of these nomograms, nomogram for predicting CSS was not verified externally due to the incompleteness of external data. Finally, the occurrence probability of different pathological types varies greatly in RPS, which caused an uneven distribution of the number of patients in different pathological types, and thus, there may be an unavoidable deviation.

## Conclusions

This study developed and validated nomograms for predicting the prognosis of patients with RPS based on a large cohort, clinicopathological and epidemiological features were integrated to predict patients 3-, 5- and 7-year OS and CSS. The nomograms have been verified both internally and externally, and provide significantly better discrimination than the AJCC TNM staging system, helping to guide monitoring and improve long-term survival outcomes, showing great potential for future clinical application. They can be used in patient consultations to provide accurate and useful information to both doctors and patients and accurately assess risk in individual patients and enhance clinical trial stratification. They also may be used in determining the proper timing for end-of-life discussions and/or hospice referrals.

### Supplementary Information

**Additional file 1: Table S1.** Results of the Multivariable Cox Models incorporating income for OS and CSS.

## Data Availability

The training datasets generated and analyzed during the current study are available in the SEER repository (https://seer.cancer.gov/); the validation datasets are available from the corresponding author on reasonable request.
