# Supplementary material for: Nomogram development and external validation for predicting overall survival and cancer-specific survival in patients with primary retroperitoneal sarcoma: a retrospective cohort study
Source: Discov Oncol. 2023 Nov 1;14:197. doi: 10.1007/s12672-023-00804-1 (PMC10620366; doi:10.1007/s12672-023-00804-1)
Supplement: Supplementary file 1 — Additional file 1: Table S1. Results of the Multivariable Cox Models incorporating income for OS and CSS. [file 12672_2023_804_MOESM1_ESM.docx]

**Table S1.** Results of the Multivariable Cox Models incorporating income for OS and CSS.

| **Variable** | **OS** | | | **CSS** | | |
| --- | --- | --- | --- | --- | --- | --- |
|  | HR | 95% CI | P | HR | 95% CI | P |
| **Age, years (OS/CSS)**  ≤55/≤54  56-78/55-78  ≥79 | -  1.50  3.01 | -  1.14 to 1.97  2.06 to 4.39 | -  <0.01  <0.001 | -  1.44  2.53 | -  1.04 to 2.00  1.52 to 4.18 | -  0.030  <0.001 |
| **Tumor size, cm (OS/CSS)**  ≤7.7/≤10.4  >7.7/>10.4 | -  3.08 | -  1.97 to 4.83 | -  <0.001 | -  2.60 | -  1.74 to 3.88 | -  <0.001 |
| **FNCLCC grade**  I  II  III | -  2.10  3.90 | -  1.46 to 3.00  2.80 to 5.42 | -  <0.001  <0.001 | -  2.69  5.28 | -  1.67 to 4.33  3.38 to 8.26 | -  <0.001  <0.001 |
| **Multifocality**  No  Yes | -  - | -  - | -  - | -  0.23 | -  0.14 to 0.38 | -  <0.001 |
| **Surgery primary site**  No  Yes | -  0.30 | -  0.20 to 0.45 | -  <0.001 | -  0.22 | -  0.14 to 0.34 | -  <0.001 |
| **Pre-/postoperative Chemotherapy**  No/unknown  Yes | -  2.02 | -  1.54 to 2.66 | -  <0.001 | -  1.93 | -  1.39 to 2.69 | -  <0.001 |
| **Median household income, dollars**  0-54999  ≥55000 | -  0.77 | -  0.54 to 1.08 | -  0.127 | -  0.87 | -  0.57 to 1.34 | -  0.526 |
| **Rural-Urban**  I  II | -  1.14 | -  0.79 to 1.66 | -  0.484 | -  1.13 | -  0.71 to 1.81 | -  0.602 |

* FNCLCC: the French Federation of Cancer Centers Sarcoma Group; Rural-Urban: I: nonmetropolitan counties not adjacent to a metropolitan area, nonmetropolitan counties adjacent to a metropolitan area and counties in metropolitan areas of less than 250 thousand population; II: counties in metropolitan areas of 250,000 to 1 million population and counties in metropolitan areas greater than or equal to 1 million population.
